# Intestinal Carnitine Status and Fatty Acid Oxidation in Response to Clofibrate and Medium-Chain Triglyceride Supplementation in Newborn Pigs

**DOI:** 10.3390/ijms24076066

**Published:** 2023-03-23

**Authors:** Brandon Pike, Jinan Zhao, Julie A. Hicks, Feng Wang, Rachel Hagen, Hsiao-Ching Liu, Jack Odle, Xi Lin

**Affiliations:** Laboratory of Developmental Nutrition, Department of Animal Sciences, North Carolina State University, Raleigh, NC 27695, USA

**Keywords:** carnitine, fatty acid oxidation, intestine, medium-chain fatty acid, newborn pigs

## Abstract

To investigate the role of peroxisome proliferator-activated receptor alpha (PPARα) in carnitine status and intestinal fatty acid oxidation in neonates, a total of 72 suckled newborn piglets were assigned into 8 dietary treatments following a 2 (±0.35% clofibrate) × 4 (diets with: succinate+glycerol (Succ), tri-valerate (TC5), tri-hexanoate (TC6), or tri-2-methylpentanoate (TMPA)) factorial design. All pigs received experimental milk diets with isocaloric energy for 5 days. Carnitine statuses were evaluated, and fatty acid oxidation was measured in vitro using [1-^14^C]-palmitic acid (1 mM) as a substrate in absence or presence of L659699 (1.6 µM), iodoacetamide (50 µM), and carnitine (1 mM). Clofibrate increased concentrations of free (41%) and/or acyl-carnitine (44% and 15%) in liver and plasma but had no effects in the intestine. The effects on carnitine status were associated with the expression of genes involved in carnitine biosynthesis, absorption, and transportation. TC5 and TMPA stimulated the increased fatty acid oxidation rate induced by clofibrate, while TC6 had no effect on the increased fatty acid oxidation induced by clofibrate (*p* > 0.05). These results suggest that dietary clofibrate improved carnitine status and increased fatty acid oxidation. Propionyl-CoA, generated from TC5 and TMPA, could stimulate the increased fatty acid oxidation rate induced by clofibrate as anaplerotic carbon sources.

## 1. Introduction

Carnitine plays a critical role in energy generation. It transports long-chain fatty acid (LCFA) into the mitochondria for oxidation and stabilizes CoA and acyl-CoA ratio in all tissues. Carnitine status and its effect on energy metabolism has been well studied in liver, muscle and plasma of newborn infants and animals [1] but has not been studied well in intestinal tissues. Newborn piglets, like human infants, have a limited capacity for carnitine biosynthesis, and carnitine in sow milk is their primary source during the suckling period [2,3]. Thus, carnitine in milk is essential for milk fats utilization and is of vital importance for neonates to obtain enough energy to thrive because milk fat is a predominant energy source for neonates at birth. To meet the energy requirement for rapid development and growth, carnitine has been added into formula for human infants [4]. The capacity of carnitine biosynthesis in liver and kidney has also been studied in numerous species including pigs [5,6,7,8]. Recently, it has been shown in rats that the enzymes needed for carnitine biosynthesis are present in other tissues, including the intestines [7]. Therefore, the role of the intestines in carnitine homeostasis should not be overlooked, especially with respect to carnitine absorption and transport in the neonate.

Carnitine and acylcarnitines are actively and/or passively absorbed into enterocytes and then transported into all other cells via the circulatory system [9,10]. Active uptake of carnitines is accomplished primarily via organic cation transporter novel family member 2 (OCTN2) and passive absorption is associated with the dose and source of carnitines and the physiological status of the intestine. The predominate role of OCTN2 in mediating the active uptake of carnitine in the small intestine has been stressed in adults [11,12,13], but very few studies in the fetus and neonates have been published.

Activation of peroxisome proliferator-activated receptor alpha (PPARα) increase expression of genes associated with carnitine synthesis such as γ-butyrobetaine hydroxylase (BBOX), N-6-trimethyllysine dioxygenase (TMLHE), and 4-trimethylaminobutyraldehyde dehydrogenase (ALDH9A1) and transport such as OCTN2 as well as carnitine concentrations in many tissues in adult animals [14,15,16]. In addition, activation of PPARα also stimulates fatty acid oxidation in liver, kidney and muscle via inducing gene expressions and increasing activities of carnitine palmitoyltransferase I (CPT I), acyl-CoA oxidase (ACOX) and malonyl-CoA decarboxylase (MCD) [17,18,19,20,21,22]. As a pharmaceutical PPARα agonist, clofibrate can activate PPARα, modulating CPT I gene expression and increasing fatty acid oxidation in pigs and other species [23,24,25,26]. As cofactor of CPT I, carnitine status is extremely important to fatty acid oxidation. However, the effect of modifying carnitine status on intestinal fatty acid oxidation has not been studied under PPARα activation in neonates.

In this study, we examined the role of PPARα activation in carnitine status and fatty acid oxidation and their relationship in the intestine using neonatal pigs as a model. We hypothesized that clofibrate-induced intestinal PPARα activation modifies carnitine status via increasing carnitine endogenous synthesis and uptake from intestine. Subsequently, the changes in carnitine status could improve fatty acid oxidation by increasing fatty acid transfer from cytoplasm to mitochondria. Furthermore, the effects of the capacity of the citric acid cycle (CAC) and the activity of ketogenic pathway on the increased fatty acid oxidation are evaluated by administration of medium-chain triglycerides (MCTs) such as tri-valerate (TC5), tri-hexanoate (TC6) and tri-2-methylpentonoate (TMPA) in the milk replacer. The medium-chain fatty acids derived from these MCTs may further accentuate fatty acid oxidation induced by PPARα activation and change the distribution of acetyl-CoA and propionyl-CoA generated by β-oxidation between the CAC and ketogenic pathways by providing anaplerotic carbon and an extra energy source.

## 2. Results

### 2.1. Carnitine Status

#### 2.1.1. Plasma Carnitine

Clofibrate had no impact on plasma (Table 1) free carnitine concentrations (*p* > 0.1), but acyl-carnitine concentration increased by 15% in clofibrate-treated pigs vs. untreated pigs (*p* < 0.05). No difference was observed between clofibrate-treated and untreated pigs for the total carnitine (free carnitine + acyl-carnitine). Diets supplemented with Succ, TC5, TC6 and TMPA had no impacts plasma free carnitine and acyl-carnitine concentrations (*p* > 0.1). There was no interactions between clofibrate and diet treatments (*p* > 0.1). The percentage of acyl-carnitine tended increase while free carnitine tended decrease (*p* = 0.064) in clofibrate treated pigs due to increased acyl-carnitine concentrations. There were no impacts of dietary treatments on the percentage of carnitines and no interactions were detected between clofibrate administration and dietary treatments.

#### 2.1.2. Liver Carnitine

Clofibrate had a significant impact on liver (Table 2) free and acyl-carnitine concentrations. Free carnitine and acyl-carnitine were 0.71 and 0.78 fold higher in pigs receiving milk supplemented with clofibrate vs. without clofibrate, respectively (*p* < 0.0001). Thus, the total carnitine in liver was increased by 1.7 fold in pigs receiving clofibrate (*p* < 0.001), but the increase did not alter the distribution between free and acyl-carnitine for either clofibrate treated or untreated pigs (*p* > 0.1). No differences were observed among the dietary MCT treatments on free, acyl- or total carnitine (*p* > 0.1) or carnitine percentage (*p* > 0.1). There were no interactions between clofibrate and the dietary treatments (*p* > 0.1).

#### 2.1.3. Intestinal Carnitine

Clofibrate and the dietary supplements had no influences on the free, acyl, or total carnitine concentration or the percentage of free carnitine (*p* > 0.1) in intestinal mucosa (Table 3). No interactions between clofibrate and the dietary treatments were not detectable (*p* > 0.1).

#### 2.1.4. Distribution of Carnitine in Plasma and Tissues

Measured carnitine concentrations showed significant differences between tissues (Table 4). On average free carnitine, acyl-carnitine and total carnitine measured in intestine tissues were 2.69, 3.27 and 3.05 fold higher than in plasma and 0.09, 1.39 and 0.7 fold higher than that in liver (*p* < 0.001), respectively. The free carnitine, acyl-carnitine and total carnitine in liver were also 1.69, 0.40 and 0.88 fold higher than in plasma, respectively. The percentage of free carnitine was higher in liver tissues than in plasma and intestine, and no difference was detected between intestine and plasma. Administration of clofibrate increased total carnitine enrichment and on average free carnitine and acyl-carnitines in plasma and tissues 54 and 19% was greater in clofibrate-treated pigs than control pigs (Table 4), but had no effect on the percentage of free and acyl-carnitine as compared to untreated pigs.

### 2.2. Fatty Acid Oxidation

#### 2.2.1. CO_2_ Production

Clofibrate (*p* < 0.05) and diet MCT treatments (*p* < 0.0001) had significant impacts on ^14^CO_2_ accumulation (nmol/(h.mg protein)) in intestinal mucosa. An interaction between clofibrate and diet MCT treatment was detected (*p* < 0.0001). The ^14^CO_2_ was on average higher in untreated pigs receiving TC6 and TMPA than those receiving Succ and TC5. Addition of clofibrate increased ^14^CO_2_ accumulation in pigs receiving diets supplemented with Succ, TC5 and TMPA, but deceased in pigs receiving the diet.

**Figure 1:**
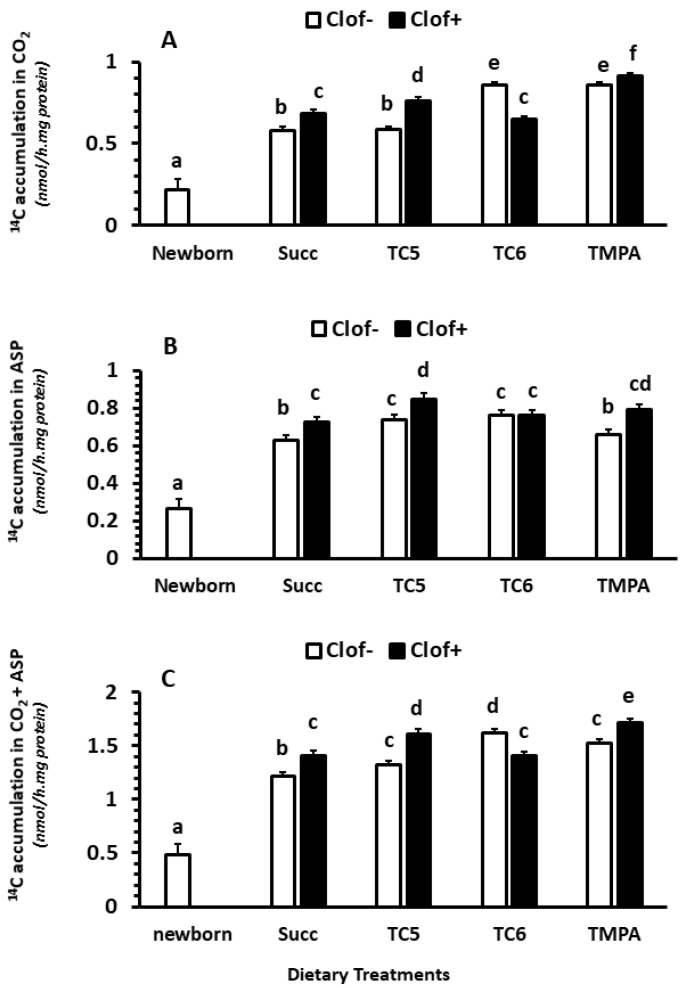
The effect of clofibrate and MCTs on intestinal fatty acid oxidation. Clof: clofibrate; Succ: Succinate + glycerol; TC5: tri-valerate; TC6: tri-hexanoate; TMPA: tri-2-methylpentonoate; ASP: acid soluble products. Data are ^14^C accumulations in CO_2_ (**A**), ASP (**B**), and CO_2_ + ASP (**C**). ^abcdef^ denotes significant (*p* < 0.05) differences between groups. Values are least square means (n = 6) ± SEM (Standard error means).

Overall, the ^14^CO_2_ accumulation from [^14^C]-palmitic acid oxidation was 3.6-fold higher in 5 d-old pigs than newborn pigs (*p* < 0.0001; Figure 1A). No interaction was observed between age and treatment with in vitro incubations (*p* > 0.1).

#### 2.2.2. Acid Soluble Products (ASP)

Clofibrate (*p* < 0.0001) and diet MCT treatments (*p* < 0.001) had significant impacts on ^14^C accumulation (nmol/(h.mg protein)) in ASP in intestinal mucosa. An interaction between clofibrate and diet MCT treatment was detected (*p* < 0.05). Clofibrate increased ASP in pigs receiving diets supplemented with Succ, TC5 and TMPA (*p* < 0.05) but not TC6 (*p* > 0.05). The highest increase in ^14^C-ASP was observed in clofibrate treated pigs that received the diet TC5. Feeding diets TC5 and TC6 to untreated pigs increased ^14^C-ASP, but the increase was not impacted by clofibrate in pigs receiving TC6 (Figure 1B).

Overall, the ^14^C accumulation in ASP from [^14^C]-palmitic acid oxidation was 2.9-fold higher in 5 d-old pigs than newborn pigs (*p* < 0.0001; Figure 1B). No interaction was observed between age and treatment with in vitro incubations (*p* > 0.1).

#### 2.2.3. Total Oxidation (CO_2_ + ASP)

Clofibrate supplementation and diet treatments significantly impacted total ^14^C-accumulation (nmol/(h.mg protein)) of palmitic acid (CO_2_ + ASP) (*p* < 0.0001) in the small intestine. An interaction between clofibrate and diet treatment was observed (*p* < 0.0001). Feeding diets TC5, TC6 and TMPA only had a greater accumulation rate compared to Succ (*p* < 0.05) in untreated pigs, while feeding diets TC6 had a greater accumulation rate compared to TC5 and TMPA in untreated pigs (*p* < 0.001). Clofibrate supplementation increased the total palmitic acid oxidation rate in pigs receiving diets with Succ, TC5 and TMPA, but clofibrate supplementation reduced the total oxidation rate by 12.5% in pigs that received the diet with TC6 (Figure 1C).

Overall, the ^14^C accumulation from [^14^C]-palmitic acid oxidation was 2.9-fold higher in 5 d-old pigs than newborn pigs (*p* < 0.0001; Figure 1C). No interaction was observed between age and treatment with in vitro incubations (*p* > 0.1).

#### 2.2.4. The Distribution between CO_2_ and ASP (% of Total Fatty Acid Oxidation)

**Figure 2:**
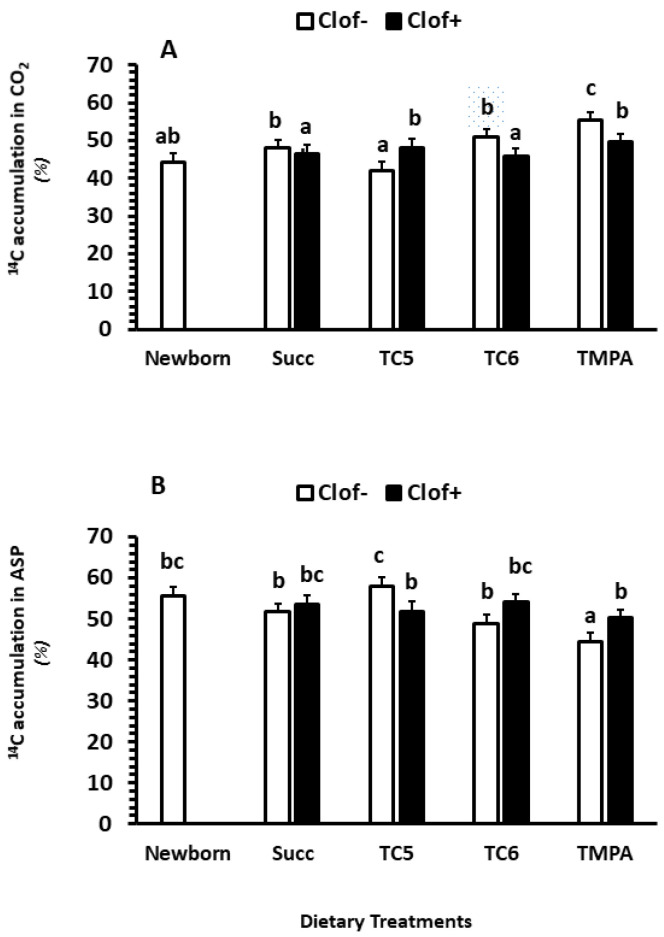
The effect of clofibrate and MCTs on the distribution (% of the total intestinal fatty acid oxidation) of CO_2_ (**A**) and ASP (**B**). Clof: clofibrate; Succ: Succinate+glycerol; TC5: tri-valerate; TC6: tri-hexanoate; TMPA: tri-2-methylpentonoate; ASP: acid soluble products. ^abc^ denotes significant (*p* < 0.05) differences between groups. Values are least square means (n = 6) ± SEM (Standard error means).

Clofibrate had no impact on the average distribution between CO_2_ and ASP (*p* > 0.1), while dietary MCT treatments had an impact on the distribution (*p* < 0.01). A significant interaction between clofibrate and diet MCT treatment was detected (*p* < 0.05). The % of CO_2_ production was on average 22% higher in untreated pigs that received diets Succ, TC6 and TMPA than TC5 (*p* < 0.0001). However, the % of CO_2_ production tended to increase by 14% in clofibrate treated pigs receiving TC5 and decrease by 11% in pigs receiving TC6 compared to the untreated pigs (*p* = 0.065). No difference was observed between clofibrate-treated and untreated pigs receiving Succ and TC6 (Figure 2A,B).

#### 2.2.5. The Effects of Carnitine, Iodoacetamide and L659699 on Fatty Acid Oxidation In Vitro

**Figure 3:**
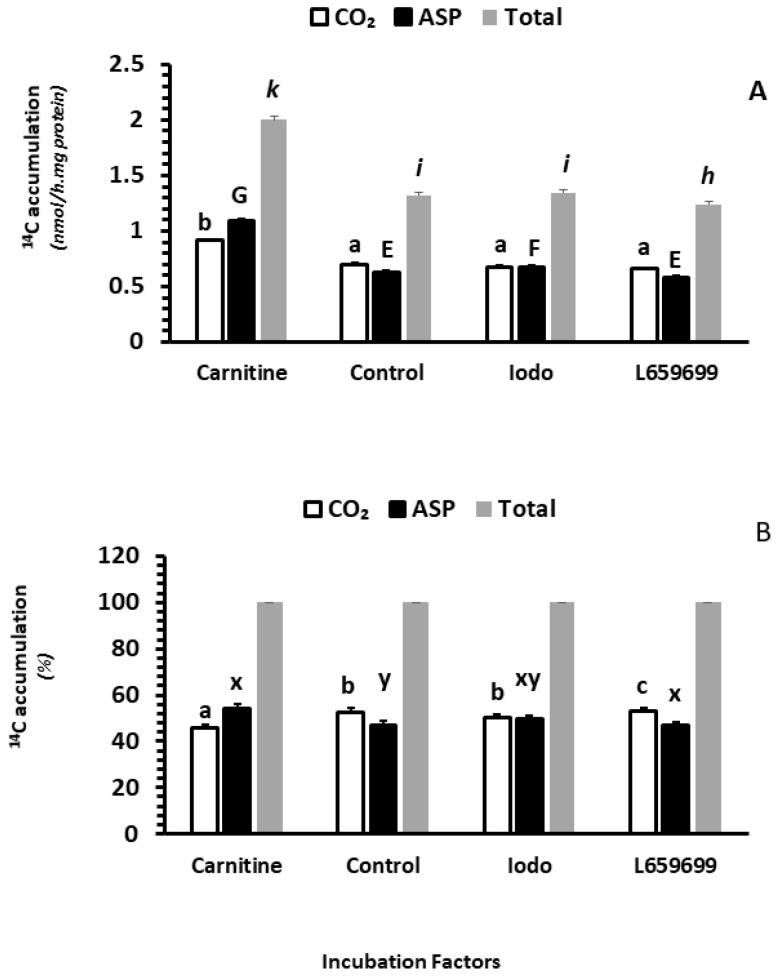
The effect of carnitine availability and ketogenic activity on intestinal fatty acid oxidation. ASP: acid soluble products. Data in (**A**) are ^14^C accumulation in CO_2_, ASP, and Total (CO_2_ + ASP). Data in (**B**) are % of CO_2_, ASP, and Total in total fatty acid oxidation. ^abc,GEF,kih^ denotes in A indicated significant (*p* < 0.05) differences between groups of CO_2_, ASP and Total, and ^abc,xy^ denotes in B indicated significant (*p* < 0.05) differences between groups of CO_2_ and ASP % of Total. Values are least square means (n = 6) ± SEM (Standard error means). Ido, iodoacetamide (50 µM), an inhibitor of acetoacetyl-CoA deacylase (AACD), L659699, an inhibitor of H hydroxymethylglutaryl-CoA synthase (MGCS).

Incubation of intestinal mucosa with carnitine in vitro significantly increased ^14^CO_2_ accumulation, while incubation with Iodoacetamide (Iodo) and L659699 significantly decreased ^14^CO_2_ accumulation (*p* < 0.05; Figure 3A). No interactions were observed between clofibrate and in vitro treatment with carnitine, Iodo and L659699 or between the dietary MCT treatment and the in vitro treatment (*p* > 0.1). Incubation of intestinal mucosa with carnitine in vitro significantly increased ^14^C accumulation in ASP compared to incubation with or without Iodo or L659699 (*p* < 0.0001). In addition, the ASP from incubation with L659699 was 12% lower than that with Iodo (*p* < 0.001; Figure 3A). No interactions were observed between clofibrate and in vitro treatment with carnitine, Iodo and L659699 and between the diet MCT treatment and the in vitro treatment (*p* > 0.1).

Overall, the palmitic acid oxidation (^14^C accumulation in CO_2_ + ASP) was increased by 52% in the intestinal mucosa incubated with carnitine in vitro compared to the control (*p* < 0.0001), while the oxidation was decreased by 6% in the incubation with L659699 compared to the control (*p* < 0.005). No difference was observed in the incubation between Iodo and control (*p* > 0.1).

Adding carnitine and L659699 (Figure 3B) in tissue incubations also changed the distribution of the ^14^C accumulation between CO_2_ and ASP. Incubation with carnitine increased the ^14^C accumulation in % of ASP (*p* < 0.0001) and incubation with L659699 increased the accumulation in CO_2_ (*p* < 0.001). Incubation with Iodo had no impact on the distribution between CO_2_ and ASP (*p* > 0.1).

### 2.3. Enzyme Activity

#### 2.3.1. Carnitine Palmitoyltransferase

Clofibrate administration significantly increased total CPT activity (*p* < 0.0001). The malonyl-CoA uninhibited enzyme, CPT II activity was increased by 17% and the malonyl-CoA inhibited enzyme (*p* < 0.01), CPT I activity was increased by 29% (*p* < 0.0001) as compared to the control group (Table 5). Dietary MCT treatment had no impacts on the activities of total CPT, CPT I and II. No interaction was observed between clofibrate and the dietary MCT treatments.

#### 2.3.2. Citrate Synthase (CS)

Clofibrate administration and the dietary MCT treatments also had no impacts on the activity of CS and mucosa protein (*p* > 0.05). No interaction was detected between clofibrate and dietary MCT treatments (Table 5).

### 2.4. Relative mRNA Abundance

#### 2.4.1. Intestinal Mucosa

No differences in expression of ACOX, CPT1A, CPT1B, BBOX, fatty acid binding protein (FABP), OCTN2, TMLHE and ALDH9A1 in intestine were observed between clofibrate –treated and untreated pigs (*p* > 1.0; Table 6). However, clofibrate treatment decreased the expression of OCTN1 (*p* < 0.05). Clofibrate supplementation also tended to decrease PPARα gene expression (*p* = 0.058). Supplementation of MCT in the diets did not impact the expression of any measured genes except for FABP (Table 6). TC5 in the diet significantly increased FABP expression compared to all other MCT diets (*p* < 0.005). There were no interactions between clofibrate and the dietary MCT treatments for all genes measured in intestinal mucosa (*p* > 0.1).

#### 2.4.2. Liver

Clofibrate administration increased hepatic expression of ALDH9A1, TMLHE and OCTN2 (*p* < 0.05) but had no impact on BBOX expression (*p* > 0.1). There were no differences in expression among the dietary Succ and MCT supplementation for the three genes measured in liver (*p* > 0.1). No interactions were detectable between clofibrate and dietary MCT treatments for all genes measured in liver (*p* > 0.1).

## 3. Discussion

### 3.1. The Effects of Clofibrate and Dietary Treatments (Succ, TC5, TC6 and TMPA) on Carnitine Status

Carnitine status has been studied extensively in numerous species including pigs [6,27,28], rodents [6,29], humans [10,30], felids [31] and canines [32], and in different tissues such as liver, muscle, kidney, heart [33,34,35], and plasma [36,37]. However, very limited information is available on carnitine status in intestine, especially in neonates. In this study, we measured carnitine and acyl-carnitine concentrations in intestine mucosa isolated from 5 d-old piglets receiving a milk replacer containing different MCTs and with or without supplementation of clofibrate, a PPARα agonist. The results showed that neither dietary MCTs nor supplementation of clofibrate had an impact on small intestinal carnitine status. The concentration of free carnitine and acyl-carnitine in intestinal mucosa of 5 d-old piglets were similar as those observed in 3-day old guinea pigs, but much higher than young guinea pigs and adult rats [38]. Concentrations measured in intestinal mucosa were also higher than in plasma and liver from fetal pigs [33], neonatal pigs [27] and those in our study. Intestinal concentrations were also higher than levels measured in skeletal muscle [39], and even higher than carnivore species (neonatal cats) [31], but similar to concentrations in the liver and muscle of neonatal dogs [32]. These results suggest that carnitine status varies from species to species and tissue to tissue.

Supplementation of clofibrate significantly increased plasma acyl-carnitine and hepatic free and acyl-carnitines (Table 1 and Table 2) although it had no impact on intestinal carnitine and acyl-carnitines. Similar results were observed previously in liver of clofibrate treated adult rats [40], and in liver and plasma of clofibrate treated hens [41]. The increase in liver but not in plasma was also observed in rats fed a diet with oxidized fat [30]. The increase in hepatic carnitines was likely associated with an increase in carnitine synthesis [42] by increased BBOX activity [43]. BBOX activity also increases with age and predominates in carnitine synthesis in pigs by 7 days of age [44]. These results suggest that the increase in carnitine in liver could be due to the increased enzyme activity the carnitine synthetic pathway. Although the activity of BBOX or the carnitine synthesis pathway was not determined in this study, we found that the expression of the gene encoding TMLHE, the first enzyme of the carnitine biosynthesis pathway, and ALDH9A1, the third enzyme in the carnitine synthesis pathway, both increased in liver of clofibrate treated pigs. An increase in ALDH9A1 expression was observed in previous studies with mice [8,30,45] but not in rats [42]. Although the effect of clofibrate on TMLHE and ALDH9A1 was not detected in rats, an increase in N-6-trimethyllysine, the carnitine metabolic precursor, was observed in previous studies with rats [46,47]. In addition to TMLHE and ALDH9A, we also found that the mRNA abundance of organic transporter-2 (OCTN2) increased in the liver of clofibrate treated pigs. This was consistent with the results observed previously in liver of mice [17,18,45,48,49], rats [50] and pigs [51,52], demonstrating that PPARα plays an important role in carnitine homeostasis by regulating OCTN2 expression. However, clofibrate administration had no impacts on the expression of BBOX in liver. A similar result was reported in rats [42] but not in mice. The expression of BBOX was increased in clofibrate treated mice [8,30,45]. These results indicate that the regulatory role and mechanism of PPARα activity in carnitine biosynthesis is likely both specie- and tissue- specific as well as impacted by physiological status such as age and development.

An association of carnitine status and regulation with development and dietary carnitine levels was observed in rats [53]. Moreover, this observation was consistent with the development and activity of the CPT system, the enzymes that require carnitine as a substrate (cofactor) for fatty acid mitochondrial transfer and oxidation. Indeed, supplementation of carnitine in the diet of young pigs resulted in a dose-dependent increase in free carnitine, acetyl and total carnitine concentrations in plasma, liver, kidney, heart, and skeletal muscle [34]. Results from a previous study in our laboratory also showed that supplementation of carnitine to sows during gestation increased the deposition of carnitine in liver, heart, and skeletal muscle of fetal piglets [33,39], and of carnitine in term fetuses in a similar study by Birkenfeld et al. [37]. It was also observed that carnitine and acyl-carnitine concentration in small intestinal mucosa in rats increased with dietary carnitine supplementation, and the concentrations of free carnitine and acyl-carnitine decreased with age from d 1 to d 29 in intestinal mucosa of developing guinea pigs [38]. Therefore, the carnitine status in intestinal mucosa in the neonate is associated with mother’s carnitine status and the carnitine concentration in the diet after birth. In our study, all pigs were from sows fed the same standard diet and pigs received the same milk replacer (the concentration of carnitine in the milk replacer was approximately 2 nmol/mg), and thus the concentration in mucosa could reflect the carnitine concentration in the milk replacer and carnitine deposited at birth and not due to the dietary supplementation of succinate and the MCFAs. Although it has been reported that dietary levels of fat, carbohydrate and protein could decrease the efficiency of carnitine absorption [54,55], the level of energy in all diets was isocaloric. In support of the observations in intestine, the concentrations of free carnitine and acyl-carnitine in plasma and liver measured in this study were not affected by the dietary energy source.

### 3.2. The Effects of Clofibrate and Dietary Treatments (Succ, TC5, TC6 and TMPA) on Fatty Acid Oxidation in Intestine

An interaction was detected between clofibrate and dietary treatment on palmitic acid oxidation in intestinal mucosa in this study. Administration of clofibrate increased the ^14^C accumulation in CO_2_, ASP and total (CO_2_ + ASP) in pigs receiving diets containing Succ, TC5 and TMPA, and the increases were consistent with the results previously observed in liver and kidney of neonatal pigs receiving dietary clofibrate [19,21,56,57,58] and by oral gavage [19,21]. This result suggests that the response of fatty acid oxidation in intestinal mucosa to PPARα activation is similar to other tissues in neonatal pigs. In previous studies, the increased fatty acid oxidation induced by clofibrate in liver and kidney was accompanied with an increase in CPT I activity and expression [19,59]. Similar to previous studies, CPT I and II activities in the intestinal mucosa were increased but changes in gene expression of CPT I and II were not detected. The same results were observed also in liver and kidney tissues [58]. A significant difference in gene expression of CPTI was observed between intestine and liver in rats during the pre and postnatal periods [59]. The expression of CPT I in intestine increased quickly after birth and reached maximum levels at 3 days, which was maintained until 12 days, while the expression in liver increased after birth and also reached maximum levels at 3 days, but then decreased quickly [59]. We examined gene expression at d 5 in this study, where the mRNA enrichment of CPT I in intestine of the piglets, like in the intestine of rat during the postnatal period, probably was at a peak value and could not be elevated any further. The difference in CPT I expression between liver and intestine was also observed in adult rats that received the PPARα agonist Wy-14643 via intraperitoneal injection [60]. Whether the lack of response of CPT I to clofibrate in intestine in this study is due to a development stage or tissue difference is not known. However, fatty acid oxidation in mitochondria is directly related to the enzyme activity that can be regulated by changing (1) the expression of CPT I, (2) the concentration of malonyl-CoA via acetyl-CoA carboxylase (ACC) and MCD; [23], and/or (3) the sensitivity of CPT I to malonyl-CoA inhibition [24]. Thus, the increase in fatty acid oxidation in intestine may be due to the increase in activity of CPT I and possibly by a reduced sensitivity to malonyl-CoA inhibition and/or malonyl-CoA concentration in the clofibrate treated pigs.

In addition, the effect of clofibrate on fatty acid oxidation in intestinal mucosa appeared to be affected by the dietary energy source. We found that dietary supplementation of MCTs increased palmitic acid oxidation by either increasing ^14^C accumulation in CO_2_ or ASP compared to diets containing Succ. This implies that dietary MCT may modify the rate of LCFA oxidation. The products of dietary TC5 from β-oxidation, acetyl-CoA and propionyl-CoA, are considered as ketogenic and anaplerotic carbon sources, respectively, [21] for potentially increasing ketone body production and CAC capacity. Compared to TC5, TC6 can only generate acetyl-CoA, and TMPA can only produce propionyl-CoA via β-oxidation. Interestingly, dietary supplementation TC5 and TMPA not only increased the rate of LCFA oxidation but also increased the stimulation of clofibrate on fatty acid oxidation compared to the Succ diet. Supplementation with TC6 increased the rate of LCFA oxidation as well but attenuated clofibrate stimulation of fatty acid oxidation (especially ^14^C accumulation in CO_2_) compared to TC5 and TMPA. This result suggests that propionyl-CoA from odd- or branch-chain MCFA, as an anaplerotic carbon source, stimulates CAC activity in intestinal tissue. Again, the effect of dietary MCTs on fatty acid oxidation with or without induction by clofibrate apparently was not associated with activity or gene expression of CPI I and II, as the dietary MCT treatments were without effect. This probably was due to the oxidation of MCFA being independent of CPT I system. Compared with CPT I, the gene expression of FABP was modified by dietary MCT, especially dietary TC5. This is similar to the results reported by Poirier et al. [61], in which a weak effect of MCFA on FABP expression was observed in the jejunum of rats. The extent of the effect might be associated with an affinity of FABP for LCFA and the assimilation of lipid in the intestine [62]. FABP as a LCFA transporter targets fatty acid to β-oxidation in liver. Significant direct interactions between FABP and PPARα in liver were reported [63,64]. As in liver, MCFA may promote intestinal LCFA to catabolic and anabolic pathways by modifying FABP gene expression.

Furthermore, we noticed that dietary supplementation of TC5, TC6 and TMAP had different effects on palmitic acid oxidation in clofibrate treated and untreated pigs, and the effects were not associated with citrate synthase. The production of CO_2_ from palmitic acid oxidation in untreated pigs receiving TC6 and TMPA was higher than in pigs receiving Succ and TC5, while the production of ASP was higher in untreated pigs receiving TC5 and TC6 than those receiving Succ and TMPA. Combining CO_2_ and ASP together, we found that the pigs not given clofibrate but receiving TC6 had the highest palmitic acid oxidation rate. This probably was related to acetyl-CoA metabolic fate and CoA availability as we speculated in kidney, in which an increased palmitic acid oxidation was only observed in pigs fed a diet with TC6 [21]. The extent of the effect might be associated with an affinity of FABP for MCFA and the structure of MCFA, because the effect of MCTs on fatty acid oxidation as the effect of even-chain MCFAs was greater than odd-chain and branch-chain MCFAs in this study. Compared to the dietary MCTs, specific transporters are needed for succinate to pass through the plasma or mitochondria membrane [65,66]. Metabolic studies showed that catabolism of ketone bodies is balanced by the ratio of succinate and succinyl-CoA [67]. The ketone body catabolic pathway uses succinyl-CoA as CoA donor, while excess of succinate inhibits ketone body utilization [67]. Therefore, the difference between dietary succinate and MCTs in CO_2_ and/or ASP is probably due to the difference in their metabolic fate.

Interestingly, not only was the total fatty acid oxidation modified but also the distribution of ^14^C accumulation (%) between CO_2_ and ASP was altered by the dietary treatments. Compared to the effect of dietary MCT, the effect of clofibrate on fatty acid oxidation did not change the % of ^14^C accumulation in CO_2_ and ASP even though the stimulation of clofibrate on fatty acid oxidation was higher from TC5 and TMPA than Succ and TC6. However, we found that TC5 reduced the % of CO_2_ and increased ASP, while TMPA increased the % of CO_2_ and decreased ASP. Because the % of CO_2_ and ASP reflects the capacity of the CAC and the activity of the ketogenic pathways, these results imply that the differences in the effects of dietary TC5 and TMPA on the CAC and ketogenic pathway were different under the current conditions. The product of β-oxidation, propionyl-CoA from TMPA could increase the CAC capacity via providing anaplerotic carbon (as discussed above), while acetyl-CoA and propionyl-CoA generated from TC5 could increase the activity of ketogenic pathway or the production of acetyl-carnitine which is part of ASP.

### 3.3. The Effects of Carnitine and Ketogenic Pathway Inhibitors on Intestinal Fatty Acid Oxidation

Milk fat is the primary energy source for neonates to grow and develop after birth. The exogenous carnitine requirement is greater for neonates than adults [68]. The effects of carnitine on fatty acid oxidation and ketogenic capacity have been well studied and documented in liver, kidney, and muscle during postnatal period [69,70,71,72,73,74]. However, to our knowledge, this is the first study to determine the effects of supplemental carnitine on intestinal fatty acid oxidation. The results from our study confirmed that supplementation of carnitine in intestinal mucosa isolated from 5-day-old pigs significantly increased fatty acid oxidation to both CO_2_ and ASP, suggesting that carnitine was a limiting factor for intestinal fatty acid oxidation. As discussed previously, carnitine status in intestinal mucosa should be associated with the dietary level of carnitine and the capability of carnitine synthesis [21,35]. Although both CO_2_ and ASP were increased by the carnitine supplementation, the % of CO_2_ was significantly reduced, suggesting that the overflow of acetyl-CoA generated from the β-oxidation can be converted to acetyl-carnitine. This result was consistent with the increase in acyl-carnitine in plasma from the clofibrate treated pigs.

Supplementation of iodoacetamide, an inhibitor of AACD for acetoacetate synthesis [75] acid oxidation or the oxidative metabolite distribution between CO_2_ and ASP. This was similar to results we obtained in kidney and liver, suggesting that the ketogenic pathway via AACD is negligible in intestine as well [21,58]. Unlike in kidney, supplementation of the L659699, an inhibitor of both mitochondrial HMGCS and cytosolic HMGCS [76], significantly reduced palmitic acid oxidation and this reduction was related to a decrease in ASP production, implying that cholesterol synthesis and/or ketogenesis might be impacted by reducing the precursor 3-hydroxy-3-methylglutaryl-CoA. Studies on ketogenic capacity in intestine are very limited, and it has been reported that the key enzyme mHMGCS is expressed in intestine of rodent species and humans [77,78,79] especially in the large intestine in which ketogenesis plays an important role in intestinal cell differentiation [80]. However, mHMGCS expression was not detected in small intestine in pigs during development [81], suggesting that the effect of L659699 on ASP observed in this study could be primarily associated with cytosolic HMGCS. In addition, the activity of HMGCS needs to be determined, as it has not been measured in either mitochondria or cytoplasm of small intestine of pigs.

## 4. Materials and Methods

### 4.1. Animal Care and Animal Protocol (Ethics Statement)

All piglets at the end of experiment were euthanized via American Veterinary Medical Association approved exsanguination while under anesthesia, and blood, liver and intestine samples were collected and processed immediately for carnitine, fatty acid oxidation and gene expression assays. All animal care/management and experimental procedures were approved by NCSU IACUC ID 16-142, approved on 14 September 2016.

Animals and Treatments: A total of 72 suckling newborn piglets were acquired from the North Carolina State University (NCSU) Swine Unit in groups of nine, each being born from the same sow. The piglets were assigned into 8 treatments within 24 h after birth following a 2 (Clofibrate (C) or No Clofibrate (NC)) × 4 (Glycerol + Succinate (Succ)), TC5, TC6, or TMPA) factorial arrangement in a randomized complete block design based on the body weight with an additional untreated piglet used as a newborn control. The average body weight was 1.31 ± 0.03 kg. The piglets were housed individually, but in sight of liter mates in a climate-controlled room at the NCSU extensive care facility.

Piglets were fed three times daily at 06:30, 14:30, and 22:30 with a standard milk replacer as described previously (Supplementary Table S1, Jinan et al., 2022) [58], with the supplements listed above at rates as follows: clofibrate at 0.35% dry matter (*w*/*w*) in 1 mL of ethanol as a vehicle, Succ at 0.48% with 0.16% glycerol, TC5 at 0.68%, TC6 at 0.64%, and TMPA at 0.64% (Supplementary Table S2). All diets were adjusted to the same energy (isocaloric) level using soybean oil. Fresh milk with supplements was prepared for each diet and stored under refrigeration for daily feedings.

### 4.2. Triglyceride Synthesis

MCTs (TC5, TC6 and TMPA) were synthesized following the same method as described previously by Heo et al. [73]. Briefly, the fatty acids were combined with glycerol and p-toluenesulfonic acid, as a catalyst, and heated at 135 °C under N2 for 72 h. After the reaction completed the synthesized triglycerides were washed to remove the unreacted substrate and the purity and quality were checked and confirmed using Waters HPLC Empower system (Milford, MA, USA) with a Lichrospher Si-100 column and standards from Sigma-Aldrich, Inc. (St. Louis, MO, USA).

### 4.3. Sample Processing

Blood samples were centrifuged at 2000× *g* for 20 min. at 4 °C and the plasma was collected and frozen in liquid nitrogen. The liver samples were cut into small pieces into liquid nitrogen directly. The frozen plasma and liver samples were transferred and stored at −80 °C until analysis. Intestinal mucosa were collected from proximal and distal ends of the small intestines at each time point in 30 cm segments, rinsed with ice-cold 0.9% NaCl solution and then opened length wise. With the segment on a smooth flat surface a glass microscope slide was used to scrape the mucosa free. The first 30 cm of the proximal end was used for fatty acid oxidation analysis and the second 30 cm was frozen in liquid nitrogen for later analysis. The first 30 cm from the distal end was frozen in liquid nitrogen as well.

### 4.4. Carnitine Analysis

Carnitine and acyl-carnitine esters in plasma, liver and intestine were analyzed following the method reported by McGarry and Foster [82] with a slight modification [33]. Briefly, free carnitine and acylcarnitines from plasma and tissues were extracted using HClO_4_, and the acidic extracts were neutralized with KOH. The precipitate of KClO_4_ was removed by centrifugation and the supernatant was used for carnitine analysis. Free carnitine and acylcarnitines in the supernatant were analyzed via the enzymatic radio-isotope method using [1-^14^C]acetyl-CoA (37 kBq/µmol) as substrate. Concentrations were corrected by blanks measured for each sample type with no acetylcarnitine transferase. All samples were run in duplicate.

### 4.5. Fatty Acid Oxidation

Fatty acid oxidation was measured using the whole fresh homogenate from the intestinal mucosa processing as previously described [57]. Assays were performed using [1-^14^C]-palmitic acid (0.5 mM, 0.28 kBq/µmol) as a substrate with and without L659699 (1.6 µM), an inhibitor of HMGCS, iodoacetamide (50 µM), an inhibitor of AACD and carnitine (1 mM). The ^14^CO_2_ trapped in the ethanolamine and ^14^C-labeled acid soluble products (ASP) were quantified using liquid scintillation (Beckman LS 6000IC, Brea, CA, USA). The homogenate protein content was analyzed following the Biuret method [83].

### 4.6. Enzyme Activity Assays

The total activity of CPT (CPT I + CPT II) was determined in mucosa homogenate without addition of malonyl-CoA, the CPT I physiological inhibitor, following the method described previously [33]. The activity of CPT II was measured by adding 2.5 mM malonyl-CoA, the inhibitor of CPT, and the activity of CPT I was calculated as the difference between total and CPT II activities. The determination was performed in a HEPES-EGTA buffer using palmitoyl-CoA and 3H-carnitine as substrate. Radioactivity in palmitoyl-carnitine was extracted by butanol and assayed via liquid scintillation as described previously [32]. Citrate synthase activity was measured in the supernatant using commercial kit from Sigma-Aldrich (St. Louis, MO, USA).

### 4.7. RNA Isolation and qPCR

Total RNA was isolated from approximately 50 mg of the frozen mucosa using Tri-reagent, following the manufacturer’s instructions (Sigma-Aldrich, Inc., St. Louis, MO, USA). Total RNA was DNase-treated using a TURBO-DNA free kit (Thermo Fisher Scientific, Waltham, MA, USA) following the manufacturer’s instructions. RNA was quantified using a Nanodrop spectrophotometer (ND-1000 ThermoFisher, Wilmington, DE, USA) and quality was assessed using agarose electrophoresis. One microgram of DNase-treated total RNA per sample was reverse transcribed using a Super Script III Reverse Transcription kit, following the manufacturer’s instructions (Thermo Fisher Scientific, Waltham, MA, USA).

Primers for qPCR (Supplemental Table S3) were designed using BLAST Primer Designer “https://www.ncbi.nlm.nih.gov/tools/primer-blast (accessed on 14 January 2018)”. Bio-Rad (Richmond, CA, USA). Each reaction contained 10ng of cDNA 500 nmol of each gene-specific forward and reverse primers, and 1X iQ SYBR Green Supermix (Bio-Rad, Hercules, CA, USA). The following PCR conditions were used: 95 °C for 5 min, followed by 40 cycles of 95 °C for 10 s, then 58 °C for 20 s. All reactions were performed in duplicate. Gene specific amplification was confirmed using melting curve analysis. The CT values were normalized to housekeeping genes (RPL9 and GAPDH) and a plate normalizer was included on each plate to account for run differences. The relative changes in gene expression (normalized to newborn pigs) were calculated from the real-time PCR data using the 2^−ΔΔCT^ method, where ΔΔC_T_ = (C_T.Target_ − C_T.GAPDH_)_age x_ − (C_T.Target_ − C_T.GAPDH_)_age0_ [83].

### 4.8. Chemicals

Clofibrate was sourced from the TCI chemical company (Portland, OR, USA). Reagents used were sourced from Sigma-Aldrich, Inc. (St. Louis, MO, USA), unless noted otherwise. [1-^14^C]-Palmitic acid, [^14^C]-acetyl-CoA and [3H]-carnitine were purchased from American Radiolabeled Chemicals, Inc. (Saint Louis, MO, USA). L659699 was sourced from Cayman Chemical Company (Ann Arbor, MI, USA). The triglycerides were synthesized in our lab with reagents from Sigma-Aldrich (St. Louis, MO, USA).

### 4.9. Statistical Analysis

Data from carnitine and acyl carnitine assays was analyzed following the GLM procedure (SAS software 9.4; Cary, NC, USA) with randomized complete block and factorial treatment structure, in which the litter was the block. Data from the measurements of fatty acid oxidation was analyzed following GLM procedure with the split-plot design. The main plot factors of clofibrate and diet treatments were in randomized blocks (litters). The treatments at tissue incubation with control, carnitine, L659699 and iodoacetamide were analyzed as subplots). The gene expressions were analyzed following the Mixed procedure with a randomized complete block design and factorial treatment arrangement. The difference between newborn and 5 d-old pigs was examined also using one-way analysis of variance. Multiple comparisons were conducted using the Tukey test. Differences were declared at a *p*-value of ≤0.05 and trends were declared when 0.05 < *p* < 0.1.

## 5. Conclusions

Activation of PPARα induced by clofibrate increased free carnitine and acylcarnitine levels in the liver and acylcarnitine in the plasma. The increases were consistent with the increased abundance of genes related to carnitine biosynthesis and transport in liver. Clofibrate supplementation also promoted intestinal fatty acid oxidation, and the promotion could be enhanced or modified by dietary MCTs, which may be associated with an increase in FABP abundance and with propionyl-CoA production, the anaplerotic carbon source to the CAC, from MCTs with odd and branch-chain fatty acids. Adequate carnitine is essential for maintaining a high fatty acid oxidation rate in the developmental intestine. The effect of activities of ketogenic pathways on fatty acid oxidation is minimal in the intestine.

## Figures and Tables

**Table 1:** The effect of clofibrate and MCTs on plasma carnitines.

	PPAR Activator		MCT								PPAR × CT
	−Clof	+Clof	SEM	*p*-Value	Succ	TC5	TC6	TMPA	SEM	*p*-Value	*p*-Value
		nmol/µL				nmol/µL					
Free Carnitine	0.078	0.090	0.009	0.362	0.075	0.091	0.096	0.075	0.012	0.497	0.847
Acyl-Carnitine	0.130 ^a^	0.153 ^b^	0.008	0.049	0.136	0.142	0.163	0.125	0.011	0.111	0.696
Total Carnitine	0.208	0.243	0.016	0.132	0.210	0.232	0.259	0.201	0.022	0.243	0.934
		%				%					
Free Carnitine	38.0	34.9	1.29	0.064	36.8	38.7	34.9	35.6	1.57	0.387	0.259
Acyl-Carnitine	62.0	65.1	1.29	0.064	63.2	61.3	65.1	64.4	1.57	0.387	0.259

PPAR: peroxisomal proliferator active-receptor; MCT: medium chain triglycerides; Clof: clofibrate; Succ: Succinate + glycerol; TC5: tri-valerate; TC6: tri-hexanoate; TMPA: tri-2-methylpentonoate. The final column is the interaction between clofibrate and MCT. ^ab^ denotes significant (*p* < 0.05) differences between groups. Values are the least square means (n = 6) ± SEM (Standard error means).

**Table 2:** The effect of clofibrate and MCTs on hepatic carnitines.

	PPAR Activator		MCT								PPAR × MCT
	−Clof	+Clof	SEM	*p*-Value	Succ	TC5	TC6	TMPA	SEM	*p*-Value	*p*-Value
	nmol/mg fresh tissue				nmol/mg fresh tissue						
Free Carnitine	0.211 ^a^	0.360 ^b^	0.015	0.001	0.269	0.275	0.309	0.287	0.020	0.552	0.841
Acyl-Carnitine	0.181 ^a^	0.323 ^b^	0.016	0.001	0.231	0.269	0.247	0.261	0.022	0.659	0.284
Total Carnitine	0.392 ^a^	0.683 ^b^	0.026	0.001	0.501	0.545	0.557	0.548	0.037	0.737	0.467
		%				%					
Free Carnitine	51.6	51.9	1.30	0.877	52.8	49.1	52.5	52.6	1.86	0.483	0.619
Acyl-Carnitine	48.4	48.1	1.30	0.877	47.2	50.9	47.5	47.3	1.86	0.483	0.619

PPAR: peroxisomal proliferator active-receptor; MCT: medium chain triglycerides; Clof: clofibrate; Succ: Succinate + glycerol; TC5: tri-valerate; TC6: tri-hexanoate; TMPA: tri-2-methylpentonoate. The final column is the interaction between clofibrate and MCT. ^ab^ denotes significant (*p* < 0.05) differences between groups. Values are the least square means (n = 6) ± SEM (Standard error means).

**Table 3:** The effect of clofibrate and MCTs on intestinal carnitines.

	PPAR Activator		MCT								PPAR × MCT
	−Clof	+Clof	SEM	*p*-Value	Succ	TC5	TC6	TMPA	SEM	*p*-Value	*p*-Value
	nmol/mg fresh tissue				nmol/mg fresh tissue						
Free Carnitine	0.291	0.323	0.015	0.134	0.296	0.317	0.301	0.313	0.020	0.878	0.351
Acyl-Carnitine	0.566	0.613	0.045	0.473	0.552	0.572	0.720	0.515	0.064	0.659	0.284
Total Carnitine	0.857	0.936	0.051	0.287	0.848	0.889	1.021	0.828	0.072	0.107	0.593
		%				%					
Free Carnitine	36.6	36.9	1.97	0.921	36.7	39.0	32.1	39.2	2.85	0.227	0.756
Acyl-Carnitine	63.4	63.1	1.97	0.901	63.3	61.0	67.9	60.8	2.85	0.227	0.756

PPAR: peroxisomal proliferator active-receptor; MCT: medium chain triglycerides; Clof: clofibrate; Succ: Succinate + glycerol; TC5: tri-valerate; TC6: tri-hexanoate; TMPA: tri-2-methylpentonoate. The final column is the interaction between clofibrate and MCT. Values are the least square means (n = 6) ± SEM (Standard error means).

**Table 4:** Distribution of carnitine in plasma and tissues.

	Clof−				Clof+				
	Plasma	Liver	Intestine		Plasma	Liver	Intestine	SEM	*p*-Value
	nmol/mg fresh tissue								
Free carnitine	0.078 ^a^	0.210 ^b^	0.292 ^c^		0.090 ^a^	0.358 ^d^	0.328 ^cd^	0.0196	0.001
Acyl-carnitine	0.128 ^a^	0.179 ^a^	0.572 ^c^		0.151 ^a^	0.318 ^b^	0.614 ^c^	0.0381	0.258
Total carnitine	0.207 ^a^	0.389 ^b^	0.865 ^d^		0.241 ^a^	0.676 ^c^	0.942 ^d^	0.0488	0.022
				%					
Free carnitine	38.21 ^a^	51.67 ^b^	36.50 ^a^		35.54 ^a^	52.12 ^b^	37.31 ^a^	2.617	0.783
Acyl-carnitine	61.79 ^b^	48.33 ^a^	63.50 ^b^		64.46 ^b^	47.88 ^a^	62.69 ^b^	2.617	0.783

PPAR: peroxisomal proliferator active-receptor; MCT: medium chain triglycerides; Clof: clofibrate; Succ: Succinate + glycerol; TC5: tri-valerate; TC6: tri-hexanoate; TMPA: tri-2-methylpentonoate. The final column is the interaction between clofibrate and MCT. ^abcd^ denotes significant (*p* < 0.05) differences between groups. Values are least square means (n = 6) ± SEM (Standard error means).

**Table 5:** The effect of clofibrate and MCTs on enzyme activity.

	PPAR Activator		MCT								PPAR × MCT
	−Clof	+Clof	SEM	*p*-Value	Succ	TC5	TC6	TMPA	SEM	*p*-Value	*p*-Value
	nmol/mg protein					nmol/mg protein					
CPT I	2.064 ^a^	2.660 ^b^	0.094	0.0001	2.418	2.368	2.199	2.462	0.135	0.503	0.626
CPT II	1.140 ^a^	1.335 ^b^	0.052	0.0120	1.168	1.310	1.234	1.234	0.074	0.629	0.718
Total CPT	3.204 ^a^	3.994 ^b^	0.105	0.0001	3.585	3.678	3.433	3.700	0.141	0.550	0.397
CS	1.705	1.568	0.617	0.128	1.751	1.621	1.648	1.525	0.088	0.340	0.490
Total protein	6.403	6.412	0.112	0.953	6.417	6.166	6.454	6.594	0.160	0.319	0.296

PPAR: peroxisomal proliferator active-receptor; MCT: medium chain triglycerides; Clof: clofibrate; Succ: Succinate + glycerol; TC5: tri-valerate; TC6: tri-hexanoate; TMPA: tri-2-methylpentonoate. CPT: carnitine palmitoyl transferase; CS: Citrate synthase; the final column is the interaction between clofibrate and MCT. ^ab^ denotes significant (*p* < 0.05) differences between groups. Values are least square means (n = 6) ± SEM (Standard error means).

**Table 6:** Gene expression measured in intestines and liver.

	NB	PPAR Activator		MCT							PPAR × MCT	
	NB	−Clof	+Clof	SEM	*p*-Value	Succ	TC5	TC6	TMPA	SEM	*p*-Value	*p*-Value
*Intestine*		Fold				Fold						
*ACOX*	1	1.52	1.52	0.086	0.318	1.50	1.62	1.64	1.59	0.121	0.861	0.259
*CPT Ia*	1	0.804	0.804	0.094	0.978	0.854	0.846	0.801	0.723	0.125	0.896	0.782
*CPT Ib*	1	0.229	0.229	0.020	0.161	0.200	0.256	0.198	0.184	0.028	0.316	0.821
*BBOX*	1	1.51	1.51	0.144	0.178	1.11	1.31	1. 44	1.62	0.205	0.354	0.845
*FABP*	1	0.623	0.623	0.056	0.944	0.438 ^a^	0.805 ^b^	0.623 ^ab^	0.629 ^ab^	0.080	0.029	0.425
*OCTN1*	1	1.363 ^b^	1.363 ^b^	0.133	0.045	1.331	1.099	1.030	1.211	0.187	0.689	0.730
*OCTN2*	1	0.686	0.686	0.095	0.308	0.538	0.657	0.616	0.657	0.135	0.918	0.222
*PPARα*	1	1.074	1.074	0.159	0.058	0.804	0.894	1.010	0.714	0.224	0.795	0.846
*TMLHE*	1	0.761	0.761	0.135	0.225	0.637	0.659	0.700	0.579	0.191	0.973	0.933
*ALDH9A1*	1	1.142	1.142	0.119	0.339	1.133	0.935	1.237	0.938	0.170	0.489	0.609
*Liver*												
*ALDH9A1*	1	1.68 ^a^	3.54 ^b^	0.39	0.003	2.27	2.03	3.04	3.11	0.550	0.430	0.606
*BBOX*	1	2.11	2.02	0.19	0.758	2.07	2.10	2.06	2.03	0.267	0.998	0.738
*TMLHE*	1	2.04 ^a^	3.70 ^b^	0.47	0.022	3.30	2.54	2.59	3.04	0.671	0.838	0.670
*OCTN2*	1	0.92 ^a^	1.90 ^b^	0.20	0.002	1.02	1.21	1.48	1.94	0.279	0.132	0.385

NB: newborn pigs, PPAR: peroxisomal proliferator active-receptor; MCT: medium chain triglycerides; Clof: clofibrate; Succ: Succinate+glycerol; TC5: tri-valerate; TC6: tri-hexanoate; TMPA: tri-2-methylpentonoate. Values are in fold change over newborn (NB). The final column is the interaction between clofibrate and MCTs. ^ab^ denotes significant (*p* < 0.05) differences between groups. Values are least square means (n = 6) ± SEM (Standard error means).

## Data Availability

Data request can be directed to the corresponding author.

## Supplementary Materials

The following supporting information can be downloaded at: https://www.mdpi.com/article/10.3390/ijms24076066/s1.
