# Supplementary material for: Intestinal Carnitine Status and Fatty Acid Oxidation in Response to Clofibrate and Medium-Chain Triglyceride Supplementation in Newborn Pigs
Source: Int J Mol Sci. 2023 Mar 23;24(7):6066. doi: 10.3390/ijms24076066 (PMC10094207; doi:10.3390/ijms24076066)
Supplement: Supplementary file 1 [file ijms-24-06066-s001.zip › ijms-2280799-supplementary.pdf]

Supplemental Table S1. Milk replacer composition and diet formulation

Ingredient	g/ 100 g of dry powder
Whey	55.57
Sodium caseinate	14.06
Whey protein concentrate	12.67
Delactosed whey	6.43
Edible lard	5.50
Dicalcium phosphate	2.76
Other#	3.01
Total	
	100.00
Calculated nutrition composition	
Energy, Kcal/g	4.23
Crude protein, %	31.25
Crude fat, %*	7.50
Lactose, %	43.18
Calcium, %	1.48
Phosphorus, %	1.32
Lysine, %	3.06
Methionine, %	1.29
Tryptophan, %	0.47
Isoleucine, %	1.80
Histidine, %	0.71
Phenylalanine, %	1.25
Threonine, %	1.62
Leucine, %	3.07
Valine, %	1.93

[#]Other, g/kg diet: mineral premix, 6.3; D/L-methionine, 6.2; potassium sorbate, 5.6; L-lysine HCl, 4.5; calcium chloride, 4.1; vitamin premix, 1.4; flavor additive, 0.6; emulsifier, 0.6; flow agent, 0.5; tetrasodium pyrophosphate, 0.3; antioxidant 0.01; Milk Specialties, Eden Prairie, MN 55344. *Whey products contributed another 2% of fat to the dry powder.

Supplemental Table S2. Experimental Dietary formulation (1 kg milk)

	Diets			
	SUC	TMPA	TC5	TC6
**Components**	Kg			
Basal dry powder	0.1280	0.1280	0.1280	0.1280
Soybean Oil	0.0259	0.0237	0.0237	0.0237
TC6				0.0064
TC5			0.0068	
T2M		0.0064		
Succinate	0.0048			
Glycerol	0.0016			
Water	0.8397	0.8419	0.8415	0.8419
Total	1	1	1	1

SUC, glycerol-succinate; TMPA, triglyceride of 2-methylpentanoic acid; TC5, triglyceride of valeric acid; TC6, triglyceride of hexanoic acid;

Supplemental Table S3. Primers used in this study with expected amplicon size post RT-qPCR. Gene Bank numbers provided to demonstrate the genes of the pig genome referenced.

	**Forward primer (5'-3'), Sen**	**Reverse primer (5'-3'), Anti**	**Amplicon Size, bp**	**NCBI (Gene Bank)**
ACOX	GGTCCATCCACGCTGTCTTA	CACGTGGGTGACTTGAGACT	119	NM_001101028.1
BBOX	AACTGGCGGTTACTTCACGG	CCACATCCCAGTCGGCATAA	91	XM_003122909.5
FABP2	CAACGAGTGGATAATGGAAAAGAGT	CCTCTTGGCTTCTACTCCTTCA	99	NM_001031780.1
CPT1A	GCTGACGATGGTTATGGGGT	TCCCGAAGCGATGAGAATCC	109	NM_001129805.1
CPT1B	GCCTGACCTATGAAGCCTCG	TGAACGAAGGCTGTGGACTC	94	NM_001007191.1
PPARα	GCTGGACGACAGTGACCTTT	AGCACATGCACGATACCCTC	117	NM_001044526.1
RXRα	GTCCTCTTCAACCCGGACTC	CTGCTCGGGGTACTTGTGTT	114	DQ279926.1
TMLHE	CGGCACACTGACACTACCTATT	AATCCATCTACCAGCAGTGTCC	104	XM_003135511.3
OCTN1	CCCACCCTGGTCAGGAACAT	GGCAGAACTCTGTTGTAAGCAC	110	NM_001145752.1
OCTN2	ATCAGATGCTCAGGGTCAAAGG	CCAGGAGGAAGGAGTCCATTTT	108	XM_013995112.1
ALD	GACTAGCTGCCGGTGTCTTC	AACTCCACAGGGCTGACATT	112	XM_021089487.1
RPL9	GCAACTGTTCGCACCATCTG	CGACGTTGATGGGGAAGTGA	109	NM_001243481.1
